# Machine Learning-Based Prediction Model of Preterm Birth Using Electronic Health Record

**DOI:** 10.1155/2022/9635526

**Published:** 2022-04-13

**Authors:** Qi Sun, Xiaoxuan Zou, Yousheng Yan, Hongguang Zhang, Shuo Wang, Yongmei Gao, Haiyan Liu, Shuyu Liu, Jianbo Lu, Ying Yang, Xu Ma

**Affiliations:** ^1^National Human Genetics Resource Center, National Research Institute for Family Planning, Beijing 100081, China; ^2^Graduate School of Peking Union Medical College, Beijing 100730, China; ^3^Haidian Maternal and Child Health Hospital, Beijing 100080, China; ^4^Beijing Obstetrics and Gynecology Hospital, Beijing 100010, China

## Abstract

**Objective:**

Preterm birth (PTB) was one of the leading causes of neonatal death. Predicting PTB in the first trimester and second trimester will help improve pregnancy outcomes. The aim of this study is to propose a prediction model based on machine learning algorithms for PTB.

**Method:**

Data for this study were reviewed from 2008 to 2018, and all the participants included were selected from a hospital in China. Six algorisms, including Naive Bayesian (NBM), support vector machine (SVM), random forest tree (RF), artificial neural networks (ANN), K-means, and logistic regression, were used to predict PTB. The receiver operating characteristic curve (ROC), accuracy, sensitivity, and specificity were used to assess the performance of the model.

**Results:**

A total of 9550 pregnant women were included in the study, of which 4775 women had PTB. A total of 4775 people were randomly selected as controls. Based on 27 weeks of gestation, the area under the curve (AUC) and the accuracy of the RF model were the highest compared with other algorithms (accuracy: 0.816; AUC = 0.885, 95% confidence interval (CI): 0.873–0.897). Meanwhile, there was positive association between the accuracy and AUC of the RF model and gestational age. Age, magnesium, fundal height, serum inorganic phosphorus, mean platelet volume, waist size, total cholesterol, triglycerides, globulins, and total bilirubin were the main influence factors of PTB.

**Conclusion:**

The results indicated that the prediction model based on the RF algorithm had a potential value to predict preterm birth in the early stage of pregnancy. The important analysis of the RF model suggested that intervention for main factors of PTB in the early stages of pregnancy would reduce the risk of PTB.

## 1. Introduction

Preterm birth (PTB) is defined as births before 37 completed weeks of gestation [1]. The PTB studied in this study was for 28–37 weeks of gestational age. Based on gestational age at delivery, PTB can be subdivided into very early preterm (<28 weeks), early preterm (28–31 weeks), moderate preterm (31–33 weeks), and late preterm (33–37 weeks) [2]. The global estimated prevalence of PTB was 11.1% (95% confidence interval [CI]: 9.1%–13.4%) [3]. The majority of PTB occurred in low- and middle-income countries [2], and the incidence of PTB in China was 6.9% in 2014 [4]. Although the incidence of premature birth was relatively low in China, PTB had a considerable impact on the health of pregnant women and children. Evidence shows that PTB was the most common cause of neonatal death and the second most frequent cause of death in children aged <5 years [5]. Further studies found that gestational age at delivery was inversely associated with the risk of neonatal morbidity and mortality [6], and about 35.00% of deaths among newborns were caused by complications of PTB [7]. Preterm neonates who survived were vulnerable to diseases, including pulmonary hypertension [8], retinopathy [9], visual and hearing impairments [10], and mental health problem [11]. Moreover, PTB not only caused death and diseases in the newborn, but also caused anxiety and depression in postpartum women [12]. Previous study showed that early screening of preterm birth pregnant women could reduce the incidence of preterm birth [13]. Therefore, a prediction model was needed to predict PTB.

Currently, numerous studies have attempted to predict preterm birth in pregnant women. Several studies supported that sonographic measurement of cervical length (CL) could be used for the prediction of PTB in the first trimester of pregnancy [14, 15], but other studies did not demonstrate the capability of CL in the screening of PTB [16, 17]. Fetal fibronectin had extensively used to predict PTB, but the sensitivity and positive predictive value of fetal fibronectin were low [18, 19]. In recent years, machine learning algorithms have been widely used in medicine with a better performance [20]. Compared with the logistic regression algorithm, the advantages of the machine learning were the ability to process higher-dimensional data and self-learn capacity [21]. Studies have shown that the use of machine learning algorithms improved the predictive accuracy of the prediction model for PTB [22, 23]. There are also some prediction models based on machine learning algorithms that have poor prediction accuracy. Weber et al. established a machine learning prediction model for preterm birth using demographic, maternal, and residency characteristics, but the predictive performance of the model was poor [24], which may be caused by inaccurate geographic information.

Inconsistent predictive power of machine learning in preterm birth. In this study, we try to use a new method to preprocess predictors. At the same time, we compared the predictive power of 6 machine learning algorithms in PTB.

## 2. Materials and Methods

### 2.1. Participants

**Figure 1:**
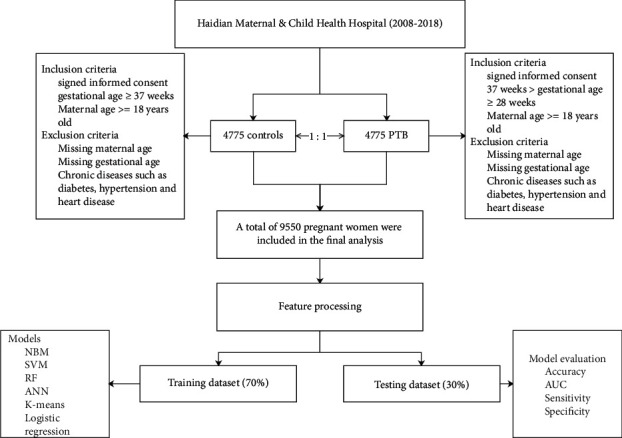
Workflow of this study.

Data for this study were reviewed from 2008 to 2018. All the participants included in this study were collected from Haidian Maternal & Child Health Hospital. The inclusion criteria of the PTB group were as follows: (1) signed informed consent; (2) gestational age between 28 and 37 weeks; and (3) maternal age older than 18 years. The exclusion criteria of the PTB group are as follows: (1) missing maternal age; (2) missing gestational age; and (3) chronic diseases such as diabetes, hypertension, and heart disease. Controls were selected from hospitals in the same period in a 1 : 1 ratio. The inclusion criteria of controls were as follows: (1) signed informed consent; (2) gestational age ≥37 weeks; and (3) maternal age ≥18 years. Exclusion criteria are as follows: (1) missing maternal age; (2) missing gestational age; (3) and chronic diseases such as diabetes, hypertension, and heart disease. The flowchart of the study is shown in Figure 1.

### 2.2. Feature Processing

Demographic factors (i.e., age), physical examination, blood test (red blood cells (RBC), white blood cell count (WBC), and plateletcrit (PCT)), urine test strip (urine pH, urine WBC, and glycosuria), and gynecological examination (bacterial vaginosis (BV), cleaning degree of vagina (CDV), and vaginal yeast infection (VYI)) were collected in our study. All participants had at least five antenatal check-ups before 27 weeks of gestation. For avoiding the overfitting of the model, variables that were measured multiple times were represented using the mean and mode, depending on the type of variable. With the increase in the gestational age, variables were more influence on the outcome. Therefore, we gave more weight to the later data. The equation is defined as

(1) $$\begin{array}{c} {\text{var}}_{\text{mean}}^{20}=\text{average}\left({\text{var}}_{{\text{week}}_{1}},{\text{var}}_{{\text{week}}_{2}},\ldots ,{\text{var}}_{{\text{week}}_{20}}\right), \end{array}$$

(2) $$\begin{array}{c} {\text{var}}_{\text{mean}}^{i}=\text{average}\left({\text{var}}_{\text{mean}}^{i-2},{\text{var}}_{{\text{week}}_{i-1}},{\text{var}}_{{\text{week}}_{i}}\right), \end{array}$$

**Figure 2:**
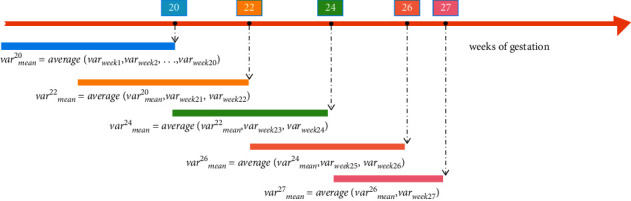
Preprocessing of variables. var_week_*i*__ is the measurement result of the variable in week i; var_mean_^20^ is the composite indicator representing the variable 20 weeks ago.

As shown in Figure 2, the variable processing process at each time point is determined by the values of the previous time point and the current time point. The dataset was divided into five datasets (20 weeks, 22 weeks, 24 weeks, 26 weeks, and 27 weeks of gestation dataset), according to the time of prenatal examination.

### 2.3. Machine Learning Algorithms

**Figure 3:**
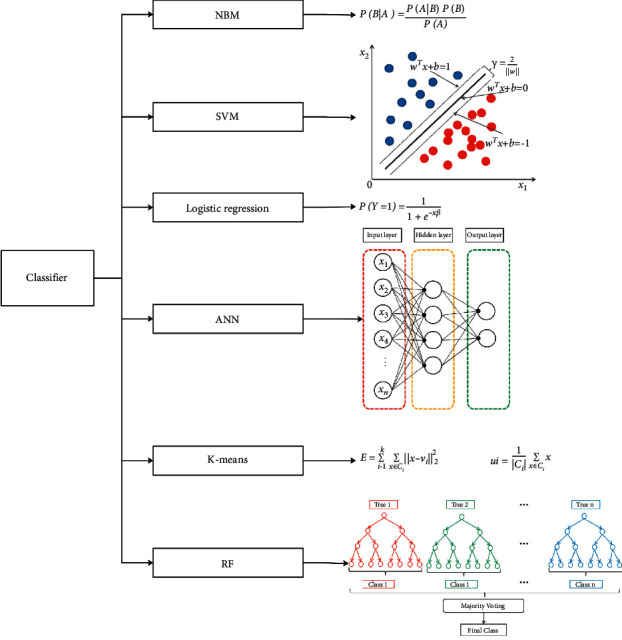
Classifiers used in this study. (1) Naive Bayesian (NBM): Naive Bayes calculates the posterior probability *P*(*B|A*) from *P*(*A*), *P*(*B*) and *P*(*B|A*); *P*(*B|A*) is the posterior probability of class B and *P*(*A*) is the prior probability of predictor A and *P*(*B*) is the prior probability of class, and *P*(*B|A*) is the probability of the predictor for the particular class. (2) Support Vector Machine (SVM); SVM outputs a hyperplane (*w*^*T*^*x*+*b*=0) that best separates the classes and has the largest separation of geometrical separations. (3) Logistic regression: The principle of logistic regression is to use a logistic function to map the results of linear regression between 0 and 1; *X* is the input features, and *β* is the weight of the features. *P*(*Y*=1) is the predicted probability of class 1. (4) Artificial Neural Networks (ANN): An artificial neural network consists of an input layer, a hidden layer, and an output layer, and its core component is an artificial neuron. Each neuron is summed by several other neurons multiplied by weights; *x*_*i*_ is the input features. (5) K-means: The K-Means algorithm minimizes the squared error for cluster *C*_*i*_; *x* is the unclassified sample, and *C*_*i*_ is the clusters, and *u*_*i*_ is the mean vector of clusters *C*_*i*_. (6) Random Forest Tree (RF): Random forest is an algorithm that integrates multiple decision trees through the Bagging idea of ensemble learning. The principle of random forest bagging is to vote the classification results of several weak classifiers to form a strong classifier.

In this study, six algorisms, including Naive Bayesian (NBM), support vector machine (SVM), random forest tree (RF), artificial neural networks (ANN), K-means, and logistic regression, were used to predict PTB (Figure 3).

### 2.4. Outcome Measure

In this study, 4 metrics were used to measure the predictive performance of the model: accuracy, area under the receiver operating characteristic curve (AUC), sensitivity, and specificity. The accuracy is the proportion of correct predictions among the total number of cases examined (1). Sensitivity refers to the test's ability to correctly detect true positive (2). Specificity relates to the test's ability to correctly detect true negative (4). AUC is a comprehensive measure of the sensitivity and specificity of the model:

(3) $$\begin{array}{c} \text{accuracy}=\frac{\text{TP}+\text{TN}}{\text{TP}+\text{TN}+\text{FP}+\text{FN}}, \end{array}$$

(4) $$\begin{array}{c} \text{sensitivity}=\frac{\text{TP}}{\text{TP}+\text{FN}}, \end{array}$$

(5) $$\begin{array}{c} \text{specificity}=\frac{\text{TN}}{\text{TN}+\text{FP}}. \end{array}$$

TP = true positive; FP = false positive; TN = true negative; FN = false negative.

### 2.5. Statistical Analysis

The Kolmogorov–Smirnov test was used to test the normality of continuous variable. If the variable satisfies normal distribution, the mean ± standard deviation was used to describe the continuous variable. Categorical variables were shown as numbers and percentages. Because our data were collected from electronic medical records, there were missing values in the dataset. Therefore, we excluded cases and variables that were missing more than 10%. For categorical variables, mode was used to fill, and for continuous variables, mean was used to fill. Comparison between the outcome groups was made by the chi-square test or Fisher's exact test for categorical variables and by the *t*-test or Wilcoxon test for continuous variables.

The dataset was randomly divided into a training set (70%) and a test set (30%). The training set was used to train the model, and the test set was used to evaluate the model. Four indicators, the area under the curve (AUC), accuracy, sensitivity, and specificity, were used to measure the performance of the model. The importance of a variable was assessed by the decreased accuracy of the model after removing the variable. The higher the decreased accuracy of the model, the more important the variable. All statistical analyses were performed in R software (version 3.5.1) using the “e1071” (Naive Bayesian algorithm and support vector machine), “randomForest” (random forest tree), and “kknn” (K-means) packages. For all analyses, if the two-tailed *P* value <0.05, the result was considered statistically significant.

### 2.6. Statement of Ethics

Ethics approval of this research was approved by the Institutional Research Review Board at National Research Institute for Family Planning and performed in accordance with the ethical standards laid down in the 1964 Declaration of Helsinki and its later amendments.

## 3. Results

### 3.1. Characteristics of Pregnant Women and Newborns

A total 9550 of pregnant women (PTB: 4775, control: 4775) were included in our study. The mean ages of the PTB group were lower than those of the control group (PTB: 29.94 ± 5.39), control: 30.72 ± 4.00, *P* < 0.001). The gestation of pregnant women was 251.19 ± 11.51 days in the case group and 274.66 ± 7.15 days in the control group (*P* < 0.001). The gravidity and parity of pregnant women in the PTB group were lower than those in the control group (all *P* < 0.001). The weight and height of newborns in the control group were higher than those in the PTB group (all *P* < 0.001). The Apgar scores (1, 5, and 10 minutes) of newborns in the control group were higher than those in the case group (all *P* < 0.001). The characteristics of pregnant women and newborns were summarized in Table 1.

### 3.2. Prenatal Testing of Pregnant Women before 27 Weeks of Gestation

In the biochemical analysis, albumin, aspartate transaminase (AST), total serum iron (TSI), magnesium (Mg), and triglycerides (TG) levels were higher in the PTB group than those in the control group (all *P* < 0.05). Meanwhile, the plasma glucose (fasting) is lower in the PTB group than that in the control group (all *P* < 0.05). Total biliary acid (TBA) and urea levels were higher in the PTB group than those in the control group (all *P* < 0.05). Platelet, intermediate cell, lymphocyte (LY), monocytes (MO), neutrophil granulocytes (NE), red blood cell distribution width-SD (RDW-SD), and WBC levels were higher in the PTB group than those in the control group. Mean cell hemoglobin (MCH), mean corpuscular hemoglobin concentration (MCHC), and platelet distribution width (PDW) were lower in the PTB group than those in the control group. Waist size, fundal height, SBP, and DBP were higher in the PTB group than those in the control group. Fetal heart rate (FHR) in the PTB group was slower than that in the control group. Urine PH was higher in the PTB group than those in the control group. Pregnant women with blood type B were found to be more common in the case group than in the control group (Table 2). The results of prenatal testing at several other time points (20, 22, 24, and 26 weeks of gestation) were described in Supplementary Tables S1–S4.

### 3.3. Performance of Prediction Models

Six algorithms (NBM, SVM, RF, ANN, K-means, and logistic regression) were used to build the model based on five datasets (20, 22, 24, 26, and 27 weeks of gestation).

**Figure 4:**
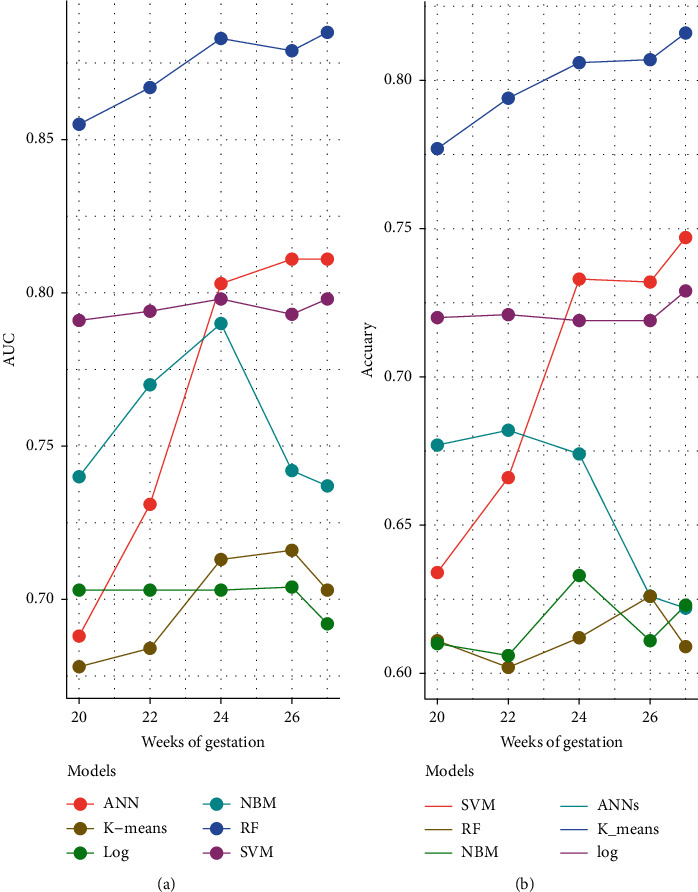
AUC (a) and accuracy (b) of models in different gestation times. (NBM: Naive Bayesian; SVM: Support Vector Machine; RF: Random Forest Tree; ANN: Artificial Neural Networks; Log: logistic regression; AUC: the area under the curve).

**Figure 5:**
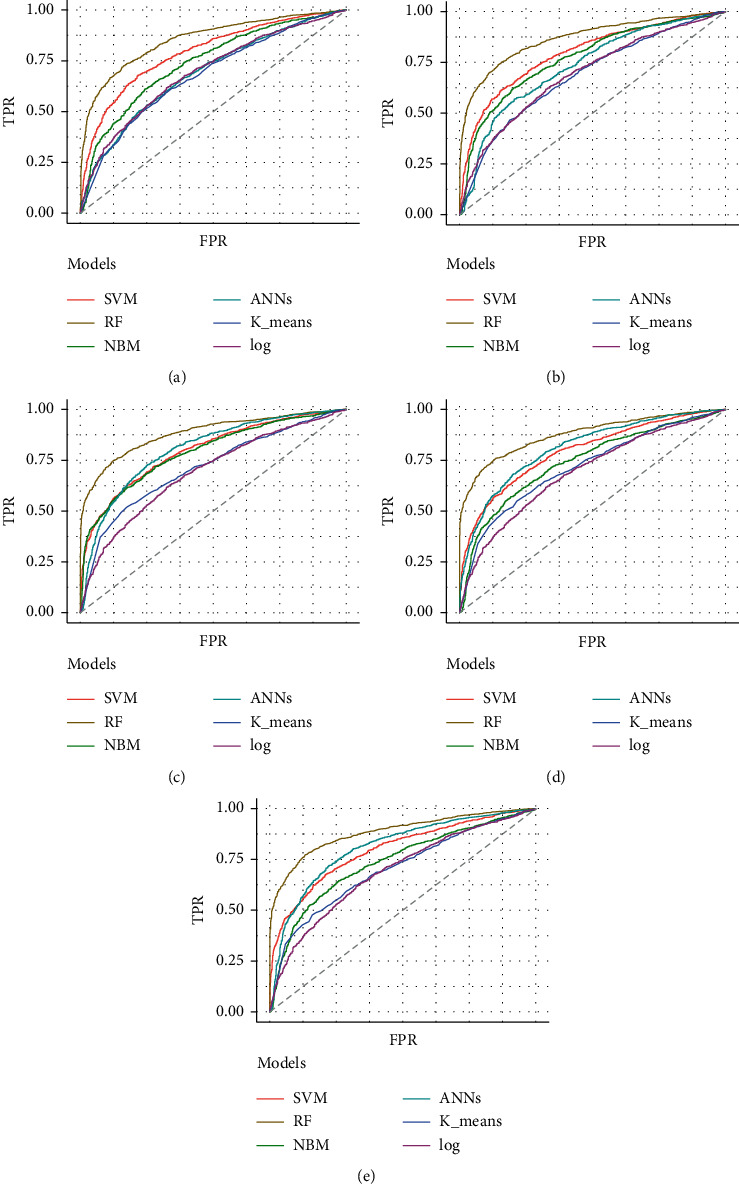
The ROC curve of the models. (a) Based on 20 weeks of gestation. (b) Based on 22 weeks of gestation. (c) Based on 24 weeks of gestation. (d) Based on 26 weeks of gestation. (e) Based on 27 weeks of gestation.

Table 3 depicts the performance of the six types of models. The results showed that the AUC and the accuracy of the RF model based on 27 weeks of gestation were the highest compared with other algorithms (accuracy: 0.816; AUC = 0.885, 95% (confidence interval) CI: 0.873–0.897). The sensitivity and specificity of the RF model based on 27 weeks of gestation were 0.751 and 0.882. Meanwhile, there was positive association between the accuracy and AUC of the RF model and gestational age (Figure 4). The sensitivity of the NBM model based on 24 weeks of gestation was 0.837, but the specificity was only 0.515. The specificity of the NBM model based on 26 weeks of gestation was 0.946, but the sensitivity was only 0.328. The receiver operating characteristic (ROC) curve of the models is shown in Figure 5.

**Figure 6:**
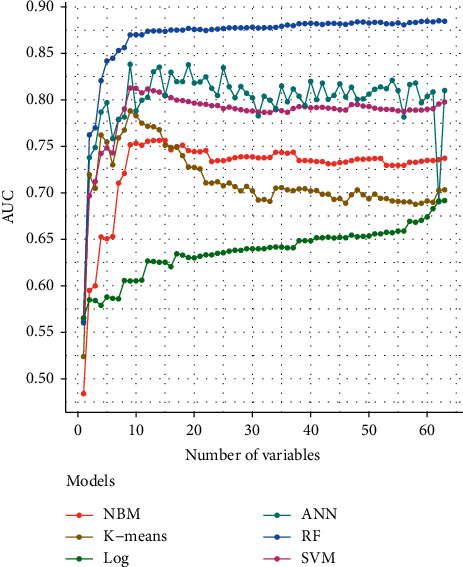
The AUC of the model increases with the number of predictors. (NBM: Naive Bayesian; SVM: Support Vector Machine; RF: Random Forest Tree; ANN: Artificial Neural Networks; Log: logistic regression; AUC: the area under the curve).

The importance analysis of the RF model found that the top 10 most important variables were age, magnesium, fundal height, serum inorganic phosphorus, mean platelet volume, waist size, total cholesterol (TC), TG, globulins, and total bilirubin (TB) (Table 4). According to the importance of variables, we gradually increase the number of predictors, and the results show that the AUC of the model also increases gradually. The AUC of the model is stable when the number of predictors increases to 15 (Figure 6).

## 4. Discussion

In this study, six algorithms were used to establish the prediction model of premature birth in the early stage of gestation. The overall prediction effect of the RF model was better than that of other models. We also found that the predictive power of the RF model increased with the increase of gestational age. Age, magnesium, fundal height, serum inorganic phosphorus, mean platelet volume, waist size, TC, TG, globulins, and TB were found to be the main influencing factors of preterm birth.

In our study, we used the data from the production inspection to build the model based on the machine learning algorithm. The prediction performance of the model was relatively good, and the cost of the model was low. Ramkumar et al. using multivariate adaptive regression splines established a prediction model based on biomarkers (including IL-1RA, TNF-*α*, angiopoietin 2, TNFRI, IL-5, MIP1*α*, IL-1*β,* and TGF-*α*), resulting in a high AUC (train set: 0.82–0.98, test set: 0.66–0.86) [25]. Teresa et al. used cervical length at admission, gestational age, amniotic fluid glucose, and interleukin-6 to establish a prediction model, resulting in a high AUC (0.86, 95% CI: 0.77–0.95) [26]. Thuy et al. found that nine cell-free RNA could be used to predict gestational age and preterm delivery, and the AUCs of preterm delivery were 0.86 in the discovery cohort and 0.81 in the validation cohort [27]. In these studies, the prediction performance of the preterm birth model was better, but another clinical test was needed and expensive. Kamala et al. used a combination of neighborhood socioeconomic status and individual status to predict preterm birth, but the AUC (0.75) of the model was relatively low [28]. Liu et al. found that cervical elastography could be used as a predictive indicator, and the AUC of the model was 0.73 [29]. The above studies used a traditional biological algorithm, such as logistic regression, to build the model, but the predictive power of the model is relatively low.

In this study, the results of the numerical experiments show that the AUC of SVM, RF, and ANN models were higher than logistic, NBM, and k-means. The possible reason for the low AUC of the NBM model is that the NBM model assumes that features are independent of each other, which is often not true in practice. For logistic regression and k-means algorithms, they were susceptible to outliers and noise that reduce prediction accuracy. For the other 3 machine algorithms, the AUC value of the RF model was the highest. The RF model is an ensemble learning method, which constructs a multitude of decision trees at training time and then sets up the trees to give the classification [30]. This ensemble strategy makes several weak classifiers form a strong classifier to improve the predictive ability of the model. In a recent study, the RF algorithm had also achieved a good predictive effect in fatty liver disease [31], suggesting that the RF algorithm had advantages in the processing of clinical electronic medical records. Moreover, we found that the prediction performance of RF was the best at 27 weeks of gestation. This may be due to alternation of biochemical indexes in pregnant women as delivery approached. The AUC of the model based on random forest in 20 weeks of gestation was 0.855 (95% CI: 0.841–0.869), suggesting that interventions could be performed before these biochemical indicators change.

In the importance analysis of the RF model, we found that age was the greatest effect on preterm birth. A case-control study showed that premature delivery was associated with greater maternal age [32]. We also found that serum magnesium had a great influence on the results of the model. A double-blind study suggested that magnesium supplementation during pregnancy is associated with a reduction in preterm delivery [33]. Maternal fundal height was found to be a valuable predictor for PTB in our study. Previous study used maternal fundal height to predict fetal weight [34], suggesting that fundal height was a good predictor for PTB. The measurement of fundal height is susceptible to measurement personnel, which may limit its clinical use. Della Rosa et al. used 9 most informative predictors to build a preterm birth prediction model, and the AUC of the model reached 0.812 [35]. Our results show that using only 15 predictions can achieve better model predictions. Considering the cost effect, this result has important implications for guiding clinical practice.

There were some limitations in our study. First, our dataset, collection from electronic medical records, and lack of some data such as smoking, drinking, family income, method of conception, medication, and fetal fibronectin. The absence of these factors may underperform our model. Second, previous studies found that the conception method has an important effect on preterm birth [36, 37], but it was not included in our model, which may affect the prediction accuracy of our model. Third, controls of the study were matched 1 : 1 from contemporaneous hospitals, which may overestimate the performance of the model and may limit the use of the model to a normal proportion of the population.

## 5. Conclusions

Our results indicated that the prediction model based on the RF algorithm had a potential value to predict preterm birth early stage of pregnancy. The RF model also found the main influence factors of PTB, suggesting that intervention in the early stages of pregnancy could decrease the risk of preterm birth.

## Figures and Tables

**Table 1:** Characteristics of mother and newborn between PTB and control group.

Variables		Control (4775)	Case (4775)	t/chi	*P*
Age, years		30.72 ± 4.00	29.94 ± 5.39	8.00	<0.001

Gestation, days		274.66 ± 7.15	251.19 ± 11.51	119.70	<0.001

Gravidity	1	3437 (0.72)	3644 (0.76)	25.08	<0.001
	2–3	1240 (0.26)	1063 (0.22)	25.08	<0.001
	>3	98 (0.02)	68 (0.01)	25.08	<0.001

Parity	1	4006 (0.84)	4176 (0.87)	24.37	<0.001
	>2	769 (0.16)	599 (0.13)	24.37	<0.001

Multiple birth	No	4763 (1.00)	4284 (0.90)	479.50	<0.001
	Yes	12 (0.00)	491 (0.10)	479.50	<0.001

Birth gender	Male	2464 (0.52)	2659 (0.56)	15.85	<0.001
	Female	2311 (0.48)	2116 (0.44)	15.85	<0.001

Birth weight, g		3410.68 ± 402.05	2691.13 ± 544.90	73.43	<0.001
Birth height, cm		50.38 ± 1.25	47.85 ± 2.82	56.81	<0.001
Apgar scores (1 min)	9.95 ± 0.71	9.70 ± 1.37	11.19	<0.001	
Apgar scores (5 min)	10.00 ± 0.66	9.82 ± 1.20	8.97	<0.001	
Apgar scores (10 min)	9.95 ± 0.54	9.77 ± 1.32	8.68	<0.001	

PTB: preterm birth.

**Table 2:** Prenatal testing of pregnant women before 27 weeks of gestation between PTB and control group.

Variables			Control (4775)	Case (4775)	t/chi	*P*
Physical examination	Waist size, cm		82.68 ± 14.19	83.30 ± 13.74	−2.17	0.030
	Fundal height, cm		20.57 ± 3.62	20.90 ± 3.84	−4.33	<0.001
	SBP, mmHg		112.09 ± 10.26	113.34 ± 10.44	−5.86	<0.001
	DBP, mmHg		69.47 ± 7.72	70.71 ± 16.09	−4.82	<0.001
	FHR, times/min		145.50 ± 3.05	146.46 ± 17.27	−3.78	<0.001
	Weight, kg		63.16 ± 9.15	63.04 ± 9.39	0.60	0.549
	Edema	No	4759 (1.00)	4747 (0.99)	2.76	0.096
		Yes	16 (0.00)	28 (0.01)		

Blood test	BG	A	1238 (0.26)	1063 (0.22)	128.27	<0.001
		B	1571 (0.33)	2106 (0.44)		
		AB	484 (0.10)	417 (0.09)		
		O	1482 (0.31)	1189 (0.25)		
	Blood RH	Ne	24 (0.01)	15 (0.00)	1.65	0.199
		Po	4751 (0.99)	4760 (1.00)		
	ALB, g/L		41.24 ± 3.46	41.45 ± 2.75	−3.20	0.001
	ALT, U/L		20.65 ± 14.05	21.02 ± 13.69	−1.28	0.199
	AST, U/L		20.91 ± 7.81	22.14 ± 7.72	−7.74	<0.001
	Glu, mmol/L		4.57 [4.25, 4.93]	4.56 [4.23, 4.72]		<0.001
	Ca, mmol/L		2.30 ± 0.14	2.31 ± 0.12	−1.36	0.174
	Cr, umol/L		50.86 ± 7.61	51.17 ± 8.25	−1.92	0.055
	DB, umol/L		1.72 [1.10, 2.30]	1.74 [1.43, 1.90]		0.594
	TSI, umol/L		17.44 ± 3.33	17.60 ± 2.63	−2.61	0.009
	GLOB, g/L		27.28 ± 3.32	27.24 ± 2.43	0.73	0.466
	Mg, mmol/L		0.87 ± 0.13	0.88 ± 0.09	−4.43	<0.001
	IP, mmol/L		1.25 ± 0.15	1.25 ± 0.12	−1.61	0.108
	TBA, umol/L		3.83 [2.90, 5.10]	4.90 [3.32, 5.01]		<0.001
	TB, umol/L		11.28 ± 3.53	11.18 ± 2.64	1.67	0.095
	CHOL, mmol/L		4.78 ± 0.73	4.80 ± 0.38	−1.66	0.096
	TP, g/L		68.76 ± 4.84	68.78 ± 3.55	−0.22	0.829
	TG, mmol/L		1.52 ± 0.54	1.58 ± 0.41	−6.57	<0.001
	Urea, mmol/L		2.80 [2.38, 3.28]	2.84 [2.40, 3.10]		0.002
	UA, umol/L		199.75 ± 40.26	198.22 ± 39.37	1.88	0.060
	BA, 10e9/L		0.01 ± 0.03	0.01 ± 0.05	−1.01	0.314
	Plt, 10e9/L		220.31 ± 48.25	224.52 ± 48.60	−4.25	<0.001
	EOS, 10e9/L		0.09 ± 0.09	0.09 ± 0.07	0.43	0.665
	Hb, g/L		117.98 ± 8.56	117.69 ± 8.59	1.62	0.105
	MID, 10e9/L		0.55 ± 0.10	0.56 ± 0.12	−4.51	<0.001
	LY, 10e9/L		1.72 ± 0.40	1.75 ± 0.41	−2.92	0.004
	MCH, pg		31.49 ± 1.91	31.33 ± 1.85	4.15	<0.001
	MCHC, g/L		344.87 ± 10.25	343.39 ± 10.52	6.95	<0.001
	MCV, fL		91.31 ± 4.72	91.24 ± 4.50	0.76	0.445
	MO, 10e9/L		0.53 ± 0.14	0.54 ± 0.14	−4.33	<0.001
	MPV, fL		8.58 ± 1.10	8.60 ± 1.09	−1.07	0.283
	NE, 10e9/L		7.23 ± 1.69	7.36 ± 1.72	−3.60	<0.001
	P-LCR, %		0.23 ± 0.05	0.23 ± 0.05	5.83	<0.001
	HCT, %		0.34 ± 0.02	0.35 ± 0.25	−1.18	0.238
	PCT, %		0.19 ± 0.04	0.19 ± 0.03	−0.23	0.819
	PDW, %		15.16 ± 2.25	14.83 ± 2.51	6.75	<0.001
	RDW-CV, %		0.16 ± 0.51	0.16 ± 0.35	0.81	0.416
	RDW-SD, fL		42.84 ± 2.46	43.45 ± 2.12	−13.07	<0.001
	RBC, 10e12L		3.76 ± 0.31	3.77 ± 0.32	−1.51	0.131
	WBC, 10e9/L		9.58 ± 1.93	9.74 ± 1.97	−3.93	<0.001

Urine test strip	Urine pH		6.67 ± 0.46	6.73 ± 0.46	−6.77	<0.001
	USG		1.02 ± 0.01	1.02 ± 0.01	4.96	<0.001
	BIL	Ne	4737 (0.99)	4749 (0.99)	1.90	0.168
		Po	38 (0.01)	26 (0.01)		
	Glycosuria	Ne	3780 (0.79)	3820 (0.80)	0.98	0.322
		Po	995 (0.21)	955 (0.20)		
	KET	Ne	4593 (0.96)	4589 (0.96)	0.03	0.873
		Po	182 (0.04)	186 (0.04)		
	Nitrituria	Ne	4728 (0.99)	4740 (0.99)	1.49	0.222
		Po	47 (0.01)	35 (0.01)		
	Blood	Ne	4322 (0.91)	4397 (0.92)	7.22	0.007
		Po	453 (0.09)	378 (0.08)		
	Proteinuria	Ne	4729 (0.99)	4698 (0.98)	7.41	0.006
		Po	46 (0.01)	77 (0.02)		
	Bilirubinuria	Ne	4758 (1.00)	4755 (1.00)	0.11	0.742
		Po	17 (0.00)	20 (0.00)		
	Urine WBC	Ne	3490 (0.73)	3475 (0.73)	0.10	0.747
		Po	1285 (0.27)	1300 (0.27)		

Gynecological examination	BV	Ne	4678 (0.98)	4719 (0.99)	10.63	0.001
		Po	97 (0.02)	56 (0.01)		
	CDV	1	854 (0.18)	975 (0.20)	60.20	<0.001
		2	2904 (0.61)	3066 (0.64)		
		3	845 (0.18)	590 (0.12)		
		4	172 (0.04)	144 (0.03)		
	VYI	Ne	4499 (0.94)	4549 (0.95)	5.05	0.025
		Po	276 (0.06)	226 (0.05)		

ALB: serum albumin; ALT: alanine transaminase; AST: aspartate transaminase; BA: basophil granulocytes; BG: blood group; BIL: urine bilirubin; Blood RH: blood RH; BV: bacterial vaginosis; Ca: total calcium; CDV: cleaning degree of vagina, The higher the value, the worse the cleanliness; CHOL: total cholesterol; Cr: creatinine; DB: direct bilirubin; DBP: diastolic blood pressure; EOS: eosinophil granulocytes; FHR: fetal heart rate; GLOB: globulins; Glu: plasma glucose (fasting); Hb: hemoglobin; HCT: hematocrit; IP: serum inorganic phosphorus; KET: urine ketone bodies; LY: lymphocytes; MCH: mean cell hemoglobin; MCHC: mean corpuscular hemoglobin concentration; MCV: mean cell volume; Mg: magnesium; MID: intermediate cell; MO: monocytes; MPV: mean platelet volume; NE: neutrophil granulocytes; PCT: plateletcrit; PDW: platelet distribution width; P-LCR: mean platelet volume; Plt: platelet count; RBC: red blood cells; RDW-CV: red blood cell distribution width-CV; RDW-SD: red blood cell distribution width-CV; SBP: systolic blood pressure; TB: total bilirubin; TBA: total biliary acid; TG: triglycerides; TP: total protein; TSI: total serum iron; UA: uric acid; Urea: urea; Urine WBC: urine white blood cell; USG: urine specific gravity; VYI: vaginal yeast infection; WBC: white blood cell count; PTB: preterm birth. Variables that are not normally distributed were expressed as p50 [p25, p75].

**Table 3:** The performance of models in the test set.

	Models	Accuracy	AUC (95% CI)	Sensitivity	Specificity
Dataset 1	SVM	0.720	0.791 (0.771–0.811)	0.710	0.731
	RF	0.777	0.861 (0.841–0.871)	0.720	0.840
	NBM	0.677	0.741 (0.721–0.761)	0.705	0.646
	ANN	0.634	0.691 (0.671–0.711)	0.687	0.576
	K-means	0.611	0.681 (0.661–0.701)	0.794	0.412
	Log	0.610	0.701 (0.681–0.721)	0.378	0.861

Dataset 2	SVM	0.721	0.791 (0.781–0.811)	0.722	0.721
	RF	0.794	0.871 (0.851–0.881)	0.756	0.832
	NBM	0.682	0.771 (0.751–0.791)	0.785	0.581
	ANN	0.666	0.731 (0.711–0.751)	0.595	0.738
	K-means	0.602	0.681 (0.671–0.701)	0.811	0.393
	Log	0.606	0.701 (0.681–0.721)	0.364	0.847

Dataset 3	SVM	0.719	0.801 (0.781–0.811)	0.695	0.743
	RF	0.806	0.881 (0.871–0.901)	0.765	0.846
	NBM	0.674	0.791 (0.771–0.811)	0.837	0.515
	ANN	0.733	0.801 (0.791–0.821)	0.741	0.726
	K-means	0.612	0.711 (0.691–0.731)	0.824	0.405
	Log	0.633	0.701 (0.681–0.721)	0.421	0.839

Dataset 4	SVM	0.719	0.791 (0.781–0.811)	0.678	0.763
	RF	0.807	0.881 (0.871–0.891)	0.743	0.875
	NBM	0.626	0.741 (0.721–0.761)	0.328	0.946
	ANN	0.732	0.811 (0.801–0.831)	0.730	0.734
	K-means	0.626	0.721 (0.701–0.741)	0.801	0.436
	Log	0.611	0.701 (0.691–0.721)	0.361	0.880

Dataset 5	SVM	0.729	0.801 (0.781–0.811)	0.685	0.773
	RF	0.816	0.891 (0.871–0.901)	0.751	0.882
	NBM	0.622	0.741 (0.721–0.761)	0.315	0.937
	ANN	0.747	0.811 (0.801–0.831)	0.730	0.763
	K-means	0.609	0.701 (0.681–0.721)	0.780	0.434
	Log	0.623	0.691 (0.671–0.711)	0.391	0.861

NBM: Naive Bayesian; SVM: Support Vector Machine; RF: Random Forest Tree; ANN: Artificial Neural Networks; Log: Logistic regression; Dataset 1: 20 weeks gestation; Dataset 2: 22 weeks gestation; Dataset 3: 24 weeks gestation; Dataset 4: 26 weeks gestation; Dataset 5: 27 weeks gestation. AUC: the area under the curve; CI: confidence interval.

**Table 4:** The top 20 importance variables of RF model.

Variables	Decreased accuracy
Age (physical examination)	0.0251
Magnesium (blood test)	0.0098
Fundal height (physical examination)	0.0077
Serum inorganic phosphorus (blood test)	0.0038
Mean platelet volume (blood test)	0.0038
Waist size (physical examination)	0.0038
Total cholesterol (blood test)	0.0035
Triglycerides (blood test)	0.0031
Globulins (blood test)	0.0024
Total bilirubin (blood test)	0.0024
Neutrophil granulocytes (blood test)	0.0024
Red blood cell distribution width-SD (blood test)	0.0024
Bacterial vaginosis (gynecological examination)	0.0021
Urine bilirubin (urine test strip)	0.0021
Urine white blood cell (urine test strip)	0.0021
Diastolic blood pressure (physical examination)	0.0014
Blood group (blood test)	0.0014
Parity (physical examination)	0.0014
Eosinophil granulocytes (blood test)	0.0010
White blood cell count (blood test)	0.0010

RF: Random Forest tree.

## Data Availability

The datasets used and analyzed during the current study are available from the corresponding author on reasonable request.

## Supplementary Materials

Supplementary Materials Table S1: prenatal testing of pregnant women before 20 weeks of gestation between the PTB group and the control group. Table S2: prenatal testing of pregnant women before 22 weeks of gestation between the PTB group and the control group. Table S3: prenatal testing of pregnant women before 24 weeks of gestation between the PTB group and the control group. Table S4: prenatal testing of pregnant women before 26 weeks of gestation between the PTB group and the control group.

## References

[1] Goldenberg R. L., Jennifer F. C., Jay D. I., Roberto R. (2008). Epidemiology and causes of preterm birth. *Lancet*.

[2] Blencowe H., Cousens S., Oestergaard M. Z., Chou D. (2012). National, regional, and worldwide estimates of preterm birth rates in the year 2010 with time trends since 1990 for selected countries: a systematic analysis and implications. *Lancet*.

[3] Beck S., Wojdyla D., Say L. (2010). The worldwide incidence of preterm birth: a systematic review of maternal mortality and morbidity. *Bulletin of the World Health Organization*.

[4] Chawanpaiboon S., Watananirun K., Lumbiganon P. (2019). Global, regional, and national estimates of levels of preterm birth in 2014: a systematic review and modelling analysis. *Lancet Global Health*.

[5] Frey H. A., Klebanoff M. A. (2016). The epidemiology, etiology, and costs of preterm birth. *Seminars in Fetal & Neonatal Medicine*.

[6] Stoll B. J., Hansen N. I., Bell E. F. (2010). Neonatal outcomes of extremely preterm infants from the NICHD Neonatal Research Network. *Pediatrics*.

[7] You D. (2015). Global, regional, and national levels and trends in under-5 mortality between 1990 and 2015, with scenario-based projections to 2030: a systematic analysis by the UN Inter-agency Group for Child Mortality Estimation. *Lancet*.

[8] Naumburg E., Soderstrom L. (2019). Increased risk of pulmonary hypertension following premature birth. *BMC Pediatrics*.

[9] Lynch A. M., Wagner, Hodges, Thevarajah T. S., McCourt, Cerda (2017). The relationship of the subtypes of preterm birth with retinopathy of prematurity. *American Journal of Obstetrics and Gynecology*.

[10] Hirvonen M. (2018). Visual and hearing impairments after preterm birth. *Pediatrics*.

[11] Luu T. M., Rehman Mian M. O., Nuyt A. M. (2017). Long-term impact of preterm birth: neurodevelopmental and physical health outcomes. *Clinics in Perinatology*.

[12] Singer L. T., Salvator A., Guo S. (1999). “Maternal psychological distress and parenting stress after the birth of a very low-birth-weight infant. *JAMA*.

[13] Ville Y., Rozenberg P. (2018). Predictors of preterm birth. *Best Practice & Research Clinical Obstetrics & Gynaecology*.

[14] Greco E., Gupta R., Syngelaki A., Poon L. C. N., Nicolaides K. H. (2012). First-trimester screening for spontaneous preterm delivery with maternal characteristics and cervical length. *Fetal Diagnosis and Therapy*.

[15] Souka A. P., Papastefanou I., Michalitsi V., Salambasis K., Chrelias C., Salamalekis G. (2011). Cervical length changes from the first to second trimester of pregnancy, and prediction of preterm birth by first-trimester sonographic cervical measurement. *Journal of Ultrasound in Medicine*.

[16] Tsikouras P., Galazios G., Zalvanos A., Bouzaki A., Athanasiadis A. (2007). Transvaginal sonographic assessment of the cervix and preterm labor. *Clinical & Experimental Obstetrics & Gynecology*.

[17] Conoscenti G., Meir Y. J., D’Ottavio G., Rustico M. A., Pinzano R., Tamaro L. F., Stampalija T. (2003). “Does cervical length at 13-15 weeks’ gestation predict preterm delivery in an unselected population?. *Ultrasound in Obstetrics and Gynecology*.

[18] Alexander D. (2003). The national Institute of Child health and human development and phenylketonuria. *Pediatrics*.

[19] Lucaroni F. (2018). Biomarkers for predicting spontaneous preterm birth: an umbrella systematic review. *Journal of Maternal-Fetal and Neonatal Medicine*.

[20] Abiodun O. I., Abiodun A. J., Dada (2018). State-of-the-art in artificial neural network applications: a survey. *Heliyon*.

[21] Ngiam K. Y., Khor L. W. (2019). Big data and machine learning algorithms for health-care delivery. *The Lancet Oncology*.

[22] Vovsha I. Predicting preterm birth is not elusive: machine learning paves the way to individual wellness.

[23] Raja R., Mukherjee I., Sarkar B. K. (2021). A machine learning-based prediction model for preterm birth in rural India. *J Healthc Eng*.

[24] Weber A., Darmstadt G. L., Gruber S., Foeller M. E., Carmichael S. L., Stevenson D. K. (2018). Application of machine-learning to predict early spontaneous preterm birth among nulliparous non-Hispanic black and white women. *Annals of Epidemiology*.

[25] Menon R. (2014). Multivariate adaptive regression splines analysis to predict biomarkers of spontaneous preterm birth. *Acta Obstetricia et Gynecologica Scandinavica*.

[26] Cobo T., Aldecoa, Herranz (2020). Development and validation of a multivariable prediction model of spontaneous preterm delivery and microbial invasion of the amniotic cavity in women with preterm labor. *American Journal of Obstetrics and Gynecology*.

[27] Ngo T. T. M., Moufarrej M. N., Rasmussen M. L. H., Soler J. C., Pan W. (2018). Noninvasive blood tests for fetal development predict gestational age and preterm delivery. *Science*.

[28] Adhikari K., Patten, Williamson (2019). Does neighborhood socioeconomic status predict the risk of preterm birth? A community-based Canadian cohort study. *BMJ Open*.

[29] Du L., Zhang, Zheng, Xie, Gu, Lin (2020). Evaluation of cervical elastography for prediction of spontaneous preterm birth in low-risk women: a prospective study. *Journal of Ultrasound in Medicine*.

[30] Ho T. K. Random decision forests.

[31] Atabaki-Pasdar N., Ohlsson, Viñuela, Frau, Millan (2020). Predicting and elucidating the etiology of fatty liver disease: a machine learning modeling and validation study in the IMI DIRECT cohorts. *PLoS Medicine*.

[32] Stylianou-Riga P., Kouis P., Kinni P., Rigas A., Papadouri T., Yiallourosx P. K. (2018). Maternal socioeconomic factors and the risk of premature birth and low birth weight in Cyprus: a case-control study. *Reproductive Health*.

[33] Spatling L., Spatling G. (1988). Magnesium supplementation in pregnancy. A double-blind study. *British Journal of Obstetrics and Gynaecology*.

[34] Anggraini D., Abdollahian M., Marion K. (2016). Accuracy assessment on prediction models for fetal weight based on maternal fundal height. *Information Technology: New Generations*.

[35] Della Rosa P. A., Miglioli, Caglioni, Tiberio, Mosser, Vignotto (2021). A hierarchical procedure to select intrauterine and extrauterine factors for methodological validation of preterm birth risk estimation. *BMC Pregnancy and Childbirth*.

[36] Cavoretto P., Candiani M., Giorgione V. (2018). Risk of spontaneous preterm birth in singleton pregnancies conceived after IVF/ICSI treatment: meta-analysis of cohort studies. *Ultrasound in Obstetrics and Gynecology*.

[37] Cavoretto P. I., Giorgione V., Sotiriadis A. (2020). IVF/ICSI treatment and the risk of iatrogenic preterm birth in singleton pregnancies: systematic review and meta-analysis of cohort studies. *Journal of Maternal-Fetal and Neonatal Medicine*.

