# Supplementary material for: Machine Learning-Based Prediction Model of Preterm Birth Using Electronic Health Record
Source: J Healthc Eng. 2022 Apr 13;2022:9635526. doi: 10.1155/2022/9635526 (PMC9020923; doi:10.1155/2022/9635526)
Supplement: Supplementary Materials — Table S1: prenatal testing of pregnant women before 20 weeks of gestation between the PTB group and the control group. Table S2: prenatal testing of pregnant women before 22 weeks of gestation between the PTB group and the control group. Table S3: prenatal testing of pregnant women before 24 weeks of gestation between the PTB group and the control group. Table S4: prenatal testing of pregnant women before 26 weeks of gestation between the PTB group and the control group. [file 9635526.f1.docx]

Table S1. Prenatal testing of pregnant women before 20 weeks of gestation between PTB group and control group

| Variables |  |  | Control (4775) | Case (4775) | t/chi | *P* |
| --- | --- | --- | --- | --- | --- | --- |
| physical examination | Waist size, cm |  | 67.67±23.86 | 69.02±22.14 | -2.85588 | 0.004 |
|  | Fundal height, cm | | 14.14±5.21 | 14.46±4.82 | -3.07749 | 0.002 |
|  | SBP, mmHg |  | 110.66±11.16 | 111.02±10.55 | -1.62633 | 0.104 |
|  | DBP, mmHg |  | 69.00±8.23 | 70.65±60.47 | -1.86715 | 0.062 |
|  | FHR, times/min |  | 148.35±3.41 | 148.99±8.96 | -4.66598 | <0.001 |
|  | Weight, Kg |  | 60.04±9.06 | 59.58±9.11 | 2.443023 | 0.015 |
|  | Edema | Ne | 3(0.00) | 0(0.00) | 1.333752 | 0.248 |
|  |  | Yes | 4772(1.00) | 4775(1.00) |  |  |
| blood test | BG | A | 1235(0.26) | 1058(0.22) | 123.4195 | <0.001 |
|  |  | B | 1593(0.33) | 2119(0.44) |  |  |
|  |  | AB | 479(0.10) | 415(0.09) |  |  |
|  |  | O | 1468(0.31) | 1183(0.25) |  |  |
|  | Blood RH | Ne | 24(0.01) | 15(0.00) | 1.647755 | 0.199 |
|  |  | Po | 4751(0.99) | 4760(1.00) |  |  |
|  | ALB, g/L |  | 41.51±3.40 | 41.76±2.65 | -4.08553 | <0.001 |
|  | ALT, U/L |  | 21.29±15.81 | 21.75±15.43 | -1.44535 | 0.148 |
|  | AST, U/L |  | 21.19±8.46 | 22.48±8.50 | -7.38182 | <0.001 |
|  | Glu, mmol/L |  | 4.61±0.55 | 4.50±0.44 | 10.81675 | <0.001 |
|  | Ca, mmol/L |  | 2.32±0.12 | 2.32±0.10 | -1.67577 | 0.094 |
|  | Cr, umol/L |  | 50.89±7.61 | 51.25±8.32 | -2.20861 | 0.027 |
|  | DB, umol/L |  | 1.78±0.96 | 1.75±0.71 | 1.499743 | 0.134 |
|  | TSI, umol/L |  | 17.52±3.34 | 17.70±2.59 | -3.05581 | 0.002 |
|  | GLOB, g/L |  | 27.33±3.31 | 27.25±2.42 | 1.284565 | 0.199 |
|  | Mg, mmol/L |  | 0.85±0.09 | 0.85±0.06 | -5.33865 | <0.001 |
|  | IP, mmol/L |  | 1.24±0.15 | 1.25±0.11 | -1.53331 | 0.125 |
|  | TBA, umol/L |  | 5.28±4.11 | 4.67±2.79 | 8.468469 | <0.001 |
|  | TB, umol/L |  | 11.39±3.56 | 11.31±2.64 | 1.21066 | 0.226 |
|  | CHOL, mmol/L |  | 4.53±0.66 | 4.46±0.34 | 6.227687 | <0.001 |
|  | TP, g/L |  | 69.08±4.76 | 69.11±3.44 | -0.4487 | 0.654 |
|  | TG, mmol/L |  | 1.35±0.47 | 1.34±0.31 | 0.426509 | 0.670 |
|  | Urea, mmol/L |  | 2.86±0.68 | 2.81±0.61 | 3.733747 | <0.001 |
|  | UA, umol/L |  | 199.04±40.05 | 197.68±39.04 | 1.67487 | 0.094 |
|  | BA, 10e9L |  | 0.01±0.03 | 0.01±0.04 | -2.66157 | 0.008 |
|  | Plt, 10e9L |  | 222.60±48.85 | 225.53±47.26 | -2.98274 | 0.003 |
|  | EOS, 10e9L |  | 0.09±0.08 | 0.09±0.07 | -0.46147 | 0.644 |
|  | Hb, g/L |  | 121.36±9.29 | 120.75±8.80 | 3.258316 | 0.001 |
|  | MID, 10e9L |  | 0.51±0.10 | 0.52±0.12 | -3.93741 | <0.001 |
|  | LY, 10e9L |  | 1.73±0.42 | 1.76±0.42 | -2.67957 | 0.007 |
|  | MCH, pg |  | 31.21±1.91 | 31.09±1.81 | 3.289866 | 0.001 |
|  | MCHC, g/L |  | 346.17±11.42 | 344.98±11.31 | 5.13148 | <0.001 |
|  | MCV, fL |  | 90.17±4.76 | 90.11±4.40 | 0.53964 | 0.589 |
|  | MO, 10e9L |  | 0.50±0.15 | 0.51±0.13 | -3.14778 | 0.002 |
|  | MPV, fL |  | 8.60±1.14 | 8.61±1.09 | -0.46292 | 0.643 |
|  | NE, 10e9L |  | 6.96±1.69 | 7.08±1.67 | -3.54683 | <0.001 |
|  | P-LCR, % |  | 0.23±0.05 | 0.22±0.04 | 4.590982 | <0.001 |
|  | HCT, % |  | 0.35±0.03 | 0.35±0.03 | 0.982391 | 0.326 |
|  | PCT, % |  | 0.19±0.04 | 0.19±0.03 | 0.550942 | 0.582 |
|  | PDW, % |  | 14.96±2.41 | 14.72±2.48 | 4.765101 | <0.001 |
|  | RDW-CV, % |  | 0.15±0.48 | 0.15±0.13 | 1.347289 | 0.178 |
|  | RDW-SD, fL |  | 42.55±2.33 | 43.07±2.10 | -11.3259 | <0.001 |
|  | RBC, 10e12L |  | 3.90±0.34 | 3.90±0.32 | 0.647837 | 0.517 |
|  | WBC, 10e9L |  | 9.29±1.93 | 9.44±1.92 | -3.85997 | <0.001 |
| urine test strip | Urine pH |  | 6.67±0.53 | 6.73±0.54 | -5.48395 | <0.001 |
|  | USG |  | 1.02±0.00 | 1.02±0.00 | 3.928287 | <0.001 |
|  | BIL | Ne | 4761(1.00) | 4767(1.00) | 1.138987 | 0.286 |
|  |  | Po | 14(0.00) | 8(0.00) |  |  |
|  | Glycosuria | Ne | 4614(0.97) | 4614(0.97) | 0 | 1.000 |
|  |  | Po | 161(0.03) | 161(0.03) |  |  |
|  | KET | Ne | 4660(0.98) | 4634(0.97) | 2.508654 | 0.113 |
|  |  | Po | 115(0.02) | 141(0.03) |  |  |
|  | Nitrituria | Ne | 4745(0.99) | 4755(1.00) | 1.628526 | 0.202 |
|  |  | Po | 30(0.01) | 20(0.00) |  |  |
|  | Blood | Ne | 4463(0.93) | 4546(0.95) | 13.1752 | <0.001 |
|  |  | Po | 312(0.07) | 229(0.05) |  |  |
|  | Proteinuria | Ne | 4760(1.00) | 4754(1.00) | 0.697072 | 0.404 |
|  |  | Po | 15(0.00) | 21(0.00) |  |  |
|  | Bilirubinuria | Ne | 4768(1.00) | 4767(1.00) | 0 | 1.000 |
|  |  | Po | 7(0.00) | 8(0.00) |  |  |
|  | Urine WBC | Ne | 4327(0.91) | 4323(0.91) | 0.01104 | 0.916 |
|  |  | Po | 448(0.09) | 452(0.09) |  |  |
| gynecological examination | BV | Ne | 4710(0.99) | 4733(0.99) | 4.574619 | 0.032 |
|  |  | Po | 65(0.01) | 42(0.01) |  |  |
|  | CDV | 1 | 938(0.20) | 1056(0.22) | 59.23378 | <0.001 |
|  |  | 2 | 3049(0.64) | 3186(0.67) |  |  |
|  |  | 3 | 690(0.14) | 468(0.10) |  |  |
|  |  | 4 | 98(0.02) | 65(0.01) |  |  |
|  | VYI | Ne | 4573(0.96) | 4612(0.97) | 4.113377 | 0.043 |
|  |  | Po | 202(0.04) | 163(0.03) |  |  |

ALB: Serum albumin; ALT: Alanine transaminase; AST: Aspartate transaminase; BA: Basophil granulocytes; BG: Blood group; BIL: Urine bilirubin; Blood RH: Blood RH; BV: Bacterial vaginosis; Ca: Total calcium; CDV: Cleaning degree of vagina; CHOL: Total cholesterol; Cr: Creatinine; DB: Direct bilirubin; DBP: Diastolic blood pressure; EOS: Eosinophil granulocytes; FHR: Fetal heart rate; GLOB: Globulins; Glu: Plasma glucose (fasting); Hb: Hemoglobin; HCT: Hematocrit; IP: Serum inorganic phosphorus; KET: Urine ketone bodies; LY: Lymphocytes; MCH: Mean cell hemoglobin; MCHC: Mean corpuscular hemoglobin concentration; MCV: Mean cell volume; Mg: Magnesium; MID: Intermediate cell; MO: Monocytes; MPV: Mean platelet volume; NE: Neutrophil granulocytes; PCT: Plateletcrit; PDW: Platelet distribution width; P-LCR: Mean platelet volume; Plt: Platelet count; RBC: Red blood cells; RDW-CV: Red blood cell distribution width-CV; RDW-SD: Red blood cell distribution width-CV; SBP: Systolic blood pressure; TB: Total bilirubin; TBA: Total biliary acid; TG: Triglycerides; TP: Total protein; TSI: Total serum iron; UA: Uric acid; Urea: Urea; Urine WBC: Urine white blood cell; USG: Urine specific gravity; VYI: Vaginal yeast infection; WBC: White blood cell count; PTB: preterm birth

Table S2. Prenatal testing of pregnant women before 22 weeks of gestation between PTB group and control group

| Variables |  |  | Control (4775) | Case (4775) | t/chi | *P* |
| --- | --- | --- | --- | --- | --- | --- |
| physical examination | Waist size, cm | | 72.70±19.72 | 73.82±18.37 | -2.89295 | 0.004 |
|  | Fundal height, cm | | 15.72±4.38 | 16.08±4.14 | -4.08516 | <0.001 |
|  | SBP, mmHg | | 111.23±10.92 | 111.81±10.50 | -2.64885 | 0.008 |
|  | DBP, mmHg | | 69.15±8.11 | 70.37±31.10 | -2.64097 | 0.008 |
|  | FHR, times/min | | 147.61±3.38 | 148.17±2.94 | -8.70475 | <0.001 |
|  | Weight, Kg | | 60.60±9.12 | 60.23±9.16 | 1.984592 | 0.047 |
|  | Edema | Ne | 6(0.00) | 1(0.00) | 2.287391 | 0.130 |
|  |  | Yes | 4769(1.00) | 4774(1.00) |  |  |
| blood test | BG | A | 1234(0.26) | 1058(0.22) | 123.9352 | <0.001 |
|  |  | B | 1591(0.33) | 2118(0.44) |  |  |
|  |  | AB | 480(0.10) | 415(0.09) |  |  |
|  |  | O | 1470(0.31) | 1184(0.25) |  |  |
|  | Blood RH | Ne | 24(0.01) | 15(0.00) | 1.647755 | 0.199 |
|  |  | Po | 4751(0.99) | 4760(1.00) |  |  |
|  | ALB, g/L |  | 41.48±3.40 | 41.68±2.65 | -3.2254 | 0.001 |
|  | ALT, U/L |  | 21.18±15.54 | 21.55±14.78 | -1.16649 | 0.243 |
|  | AST, U/L |  | 21.15±8.38 | 22.37±8.15 | -7.24608 | <0.001 |
|  | Glu, mmol/L | | 4.61±0.55 | 4.50±0.44 | 10.9996 | <0.001 |
|  | Ca, mmol/L | | 2.32±0.13 | 2.32±0.10 | -1.72771 | 0.084 |
|  | Cr, umol/L |  | 50.87±7.60 | 51.22±8.32 | -2.10759 | 0.035 |
|  | DB, umol/L | | 1.77±0.95 | 1.74±0.71 | 1.866126 | 0.062 |
|  | TSI, umol/L | | 17.52±3.33 | 17.67±2.57 | -2.53218 | 0.011 |
|  | GLOB, g/L |  | 27.33±3.30 | 27.26±2.41 | 1.179138 | 0.238 |
|  | Mg, mmol/L | | 0.85±0.10 | 0.85±0.07 | -4.09809 | <0.001 |
|  | IP, mmol/L | | 1.24±0.15 | 1.25±0.11 | -1.69273 | 0.091 |
|  | TBA, umol/L | | 5.28±4.11 | 4.68±2.70 | 8.401439 | <0.001 |
|  | TB, umol/L | | 11.37±3.55 | 11.27±2.63 | 1.641652 | 0.101 |
|  | CHOL, mmol/L | | 4.60±0.65 | 4.58±0.33 | 2.520248 | 0.012 |
|  | TP, g/L |  | 69.04±4.77 | 69.04±3.43 | 0.071403 | 0.943 |
|  | TG, mmol/L | | 1.39±0.49 | 1.41±0.30 | -1.73104 | 0.083 |
|  | Urea, mmol/L | | 2.86±0.68 | 2.81±0.62 | 3.706724 | <0.001 |
|  | UA, umol/L | | 199.09±39.89 | 197.72±39.06 | 1.693647 | 0.090 |
|  | BA, 10e9L |  | 0.01±0.03 | 0.01±0.05 | -2.73244 | 0.006 |
|  | Plt, 10e9L |  | 222.41±48.79 | 225.70±47.50 | -3.33926 | <0.001 |
|  | EOS, 10e9L | | 0.09±0.08 | 0.09±0.07 | -0.77785 | 0.437 |
|  | Hb, g/L |  | 120.50±9.19 | 119.90±8.80 | 3.239461 | 0.001 |
|  | MID, 10e9L | | 0.52±0.10 | 0.53±0.12 | -4.37478 | <0.001 |
|  | LY, 10e9L |  | 1.73±0.41 | 1.76±0.41 | -2.99064 | 0.003 |
|  | MCH, pg |  | 31.27±1.90 | 31.14±1.82 | 3.593132 | <0.001 |
|  | MCHC, g/L | | 346.01±11.19 | 344.59±11.15 | 6.203013 | <0.001 |
|  | MCV, fL |  | 90.39±4.72 | 90.36±4.44 | 0.256819 | 0.797 |
|  | MO, 10e9L | | 0.51±0.15 | 0.52±0.13 | -3.581 | <0.001 |
|  | MPV, fL |  | 8.60±1.13 | 8.60±1.08 | -0.30588 | 0.760 |
|  | NE, 10e9L |  | 7.03±1.69 | 7.17±1.67 | -4.03214 | <0.001 |
|  | P-LCR, % |  | 0.23±0.05 | 0.22±0.04 | 4.979118 | <0.001 |
|  | HCT, % |  | 0.35±0.03 | 0.35±0.03 | 0.542849 | 0.587 |
|  | PCT, % |  | 0.19±0.04 | 0.19±0.03 | 0.554328 | 0.579 |
|  | PDW, % |  | 14.98±2.38 | 14.74±2.48 | 4.835062 | <0.001 |
|  | RDW-CV, % | | 0.15±0.45 | 0.15±0.13 | 1.22271 | 0.221 |
|  | RDW-SD, fL | | 42.70±2.36 | 43.27±2.11 | -12.5644 | <0.001 |
|  | RBC, 10e12L | | 3.87±0.33 | 3.86±0.32 | 0.385072 | 0.700 |
|  | WBC, 10e9L | | 9.36±1.92 | 9.54±1.92 | -4.38593 | <0.001 |
| urine test strip | Urine pH |  | 6.70±0.51 | 6.76±0.52 | -6.19441 | <0.001 |
|  | USG |  | 1.02±0.00 | 1.02±0.00 | 5.190045 | <0.001 |
|  | BIL | Ne | 4758(1.00) | 4762(1.00) | 0.300945 | 0.583 |
|  |  | Po | 17(0.00) | 13(0.00) |  |  |
|  | Glycosuria | Ne | 4485(0.94) | 4485(0.94) | 0 | 1.000 |
|  |  | Po | 290(0.06) | 290(0.06) |  |  |
|  | KET | Ne | 4641(0.97) | 4626(0.97) | 0.71373 | 0.398 |
|  |  | Po | 134(0.03) | 149(0.03) |  |  |
|  | Nitrituria | Ne | 4739(0.99) | 4753(1.00) | 2.931598 | 0.087 |
|  |  | Po | 36(0.01) | 22(0.00) |  |  |
|  | Blood | Ne | 4423(0.93) | 4511(0.94) | 13.13455 | <0.001 |
|  |  | Po | 352(0.07) | 264(0.06) |  |  |
|  | Proteinuria | Ne | 4756(1.00) | 4741(0.99) | 3.718751 | 0.054 |
|  |  | Po | 19(0.00) | 34(0.01) |  |  |
|  | Bilirubinuria | Ne | 4767(1.00) | 4765(1.00) | 0.05566 | 0.813 |
|  |  | Po | 8(0.00) | 10(0.00) |  |  |
|  | Urine WBC | Ne | 4115(0.86) | 4092(0.86) | 0.419361 | 0.517 |
|  |  | Po | 660(0.14) | 683(0.14) |  |  |
| gynecological examination | BV | Ne | 4703(0.98) | 4732(0.99) | 6.900486 | 0.009 |
|  |  | Po | 72(0.02) | 43(0.01) |  |  |
|  | CDV | 1 | 918(0.19) | 1041(0.22) | 67.03055 | <0.001 |
|  |  | 2 | 3006(0.63) | 3162(0.66) |  |  |
|  |  | 3 | 742(0.16) | 490(0.10) |  |  |
|  |  | 4 | 109(0.02) | 82(0.02) |  |  |
|  | VYI | Ne | 4554(0.95) | 4598(0.96) | 4.847761 | 0.028 |
|  |  | Po | 221(0.05) | 177(0.04) |  |  |

ALB: Serum albumin; ALT: Alanine transaminase; AST: Aspartate transaminase; BA: Basophil granulocytes; BG: Blood group; BIL: Urine bilirubin; Blood RH: Blood RH; BV: Bacterial vaginosis; Ca: Total calcium; CDV: Cleaning degree of vagina; CHOL: Total cholesterol; Cr: Creatinine; DB: Direct bilirubin; DBP: Diastolic blood pressure; EOS: Eosinophil granulocytes; FHR: Fetal heart rate; GLOB: Globulins; Glu: Plasma glucose (fasting); Hb: Hemoglobin; HCT: Hematocrit; IP: Serum inorganic phosphorus; KET: Urine ketone bodies; LY: Lymphocytes; MCH: Mean cell hemoglobin; MCHC: Mean corpuscular hemoglobin concentration; MCV: Mean cell volume; Mg: Magnesium; MID: Intermediate cell; MO: Monocytes; MPV: Mean platelet volume; NE: Neutrophil granulocytes; PCT: Plateletcrit; PDW: Platelet distribution width; P-LCR: Mean platelet volume; Plt: Platelet count; RBC: Red blood cells; RDW-CV: Red blood cell distribution width-CV; RDW-SD: Red blood cell distribution width-CV; SBP: Systolic blood pressure; TB: Total bilirubin; TBA: Total biliary acid; TG: Triglycerides; TP: Total protein; TSI: Total serum iron; UA: Uric acid; Urea: Urea; Urine WBC: Urine white blood cell; USG: Urine specific gravity; VYI: Vaginal yeast infection; WBC: White blood cell count; PTB: preterm birth

Table S3. Prenatal testing of pregnant women before 24 weeks of gestation between PTB group and control group

| Variables |  |  | Control (4775) | Case (4775) | t/chi | *P* |
| --- | --- | --- | --- | --- | --- | --- |
| physical examination | Waist size, cm | | 78.39±16.15 | 79.34±14.73 | -2.9953 | 0.003 |
|  | Fundal height, cm | | 18.13±3.70 | 18.51±3.57 | -5.05186 | <0.001 |
|  | SBP, mmHg | | 112.20±10.66 | 113.03±10.39 | -3.83849 | <0.001 |
|  | DBP, mmHg | | 69.47±7.97 | 70.65±17.07 | -4.32847 | <0.001 |
|  | FHR, times/min | | 146.49±3.32 | 147.06±2.96 | -8.83886 | <0.001 |
|  | Weight, Kg | | 61.74±9.16 | 61.57±9.21 | 0.940618 | 0.347 |
|  | Edema | Ne | 9(0.00) | 10(0.00) | 0 | 1.000 |
|  |  | Yes | 4766(1.00) | 4765(1.00) |  |  |
| blood test | BG | A | 1237(0.26) | 1063(0.22) | 128.6701 | <0.001 |
|  |  | B | 1572(0.33) | 2108(0.44) |  |  |
|  |  | AB | 484(0.10) | 415(0.09) |  |  |
|  |  | O | 1482(0.31) | 1189(0.25) |  |  |
|  | Blood RH | Ne | 24(0.01) | 15(0.00) | 1.647755 | 0.199 |
|  |  | Po | 4751(0.99) | 4760(1.00) |  |  |
|  | ALB, g/L |  | 41.37±3.45 | 41.57±2.68 | -3.18014 | 0.001 |
|  | ALT, U/L |  | 20.91±14.64 | 21.30±14.14 | -1.29477 | 0.195 |
|  | AST, U/L |  | 21.02±8.04 | 22.26±7.88 | -7.63083 | <0.001 |
|  | Glu, mmol/L | | 4.61±0.55 | 4.50±0.44 | 10.70768 | <0.001 |
|  | Ca, mmol/L | | 2.31±0.13 | 2.31±0.11 | -1.58481 | 0.113 |
|  | Cr, umol/L |  | 50.87±7.64 | 51.21±8.28 | -2.07116 | 0.038 |
|  | DB, umol/L | | 1.76±0.95 | 1.73±0.72 | 1.956434 | 0.050 |
|  | TSI, umol/L | | 17.48±3.34 | 17.65±2.60 | -2.79613 | 0.005 |
|  | GLOB, g/L |  | 27.32±3.32 | 27.26±2.42 | 0.980406 | 0.327 |
|  | Mg, mmol/L | | 0.85±0.12 | 0.86±0.07 | -3.63417 | <0.001 |
|  | IP, mmol/L | | 1.24±0.15 | 1.25±0.12 | -1.72549 | 0.084 |
|  | TBA, umol/L | | 5.29±4.13 | 4.69±2.70 | 8.37517 | <0.001 |
|  | TB, umol/L | | 11.32±3.55 | 11.22±2.64 | 1.655207 | 0.098 |
|  | CHOL, mmol/L | | 4.70±0.68 | 4.71±0.33 | -0.37608 | 0.707 |
|  | TP, g/L |  | 68.93±4.81 | 68.92±3.46 | 0.0214 | 0.983 |
|  | TG, mmol/L | | 1.45±0.51 | 1.49±0.31 | -4.00983 | <0.001 |
|  | Urea, mmol/L | | 2.86±0.68 | 2.81±0.62 | 3.752325 | <0.001 |
|  | UA, umol/L | | 199.35±40.16 | 197.88±39.07 | 1.815985 | 0.069 |
|  | BA, 10e9L |  | 0.01±0.03 | 0.01±0.05 | -1.66779 | 0.095 |
|  | Plt, 10e9L |  | 221.85±48.53 | 225.51±48.39 | -3.69266 | <0.001 |
|  | EOS, 10e9L | | 0.09±0.08 | 0.09±0.07 | -0.563 | 0.573 |
|  | Hb, g/L |  | 119.24±9.04 | 118.69±8.77 | 2.999388 | 0.003 |
|  | MID, 10e9L | | 0.54±0.10 | 0.55±0.11 | -4.70634 | <0.001 |
|  | LY, 10e9L |  | 1.72±0.41 | 1.75±0.42 | -2.80188 | 0.005 |
|  | MCH, pg |  | 31.38±1.90 | 31.25±1.83 | 3.584113 | <0.001 |
|  | MCHC, g/L | | 345.56±10.89 | 344.02±10.96 | 6.906036 | <0.001 |
|  | MCV, fL |  | 90.82±4.71 | 90.83±4.48 | -0.11618 | 0.908 |
|  | MO, 10e9L | | 0.52±0.15 | 0.53±0.13 | -4.01641 | <0.001 |
|  | MPV, fL |  | 8.58±1.11 | 8.59±1.09 | -0.65774 | 0.511 |
|  | NE, 10e9L |  | 7.15±1.70 | 7.30±1.72 | -4.25196 | <0.001 |
|  | P-LCR, % |  | 0.23±0.05 | 0.22±0.04 | 5.536427 | <0.001 |
|  | HCT, % |  | 0.35±0.03 | 0.35±0.02 | 0.0412 | 0.967 |
|  | PCT, % |  | 0.19±0.04 | 0.19±0.03 | 0.349656 | 0.727 |
|  | PDW, % |  | 15.06±2.32 | 14.79±2.51 | 5.42837 | <0.001 |
|  | RDW-CV, % | | 0.16±0.55 | 0.15±0.10 | 2.022879 | 0.043 |
|  | RDW-SD, fL | | 42.82±2.40 | 43.43±2.14 | -12.9779 | <0.001 |
|  | RBC, 10e12L | | 3.81±0.33 | 3.81±0.32 | 0.161611 | 0.872 |
|  | WBC, 10e9L | | 9.49±1.94 | 9.67±1.97 | -4.53269 | <0.001 |
| urine test strip | Urine pH |  | 6.71±0.49 | 6.76±0.48 | -5.25423 | <0.001 |
|  | USG |  | 1.02±0.01 | 1.02±0.00 | 5.293915 | <0.001 |
|  | BIL | Ne | 4751(0.99) | 4756(1.00) | 0.373776 | 0.541 |
|  |  | Po | 24(0.01) | 19(0.00) |  |  |
|  | Glycosuria | Ne | 4214(0.88) | 4211(0.88) | 0.00403 | 0.949 |
|  |  | Po | 561(0.12) | 564(0.12) |  |  |
|  | KET | Ne | 4624(0.97) | 4616(0.97) | 0.163368 | 0.686 |
|  |  | Po | 151(0.03) | 159(0.03) |  |  |
|  | Nitrituria | Ne | 4736(0.99) | 4746(0.99) | 1.199719 | 0.273 |
|  |  | Po | 39(0.01) | 29(0.01) |  |  |
|  | Blood | Ne | 4376(0.92) | 4449(0.93) | 7.737767 | 0.005 |
|  |  | Po | 399(0.08) | 326(0.07) |  |  |
|  | Proteinuria | Ne | 4741(0.99) | 4729(0.99) | 1.525277 | 0.217 |
|  |  | Po | 34(0.01) | 46(0.01) |  |  |
|  | Bilirubinuria | Ne | 4760(1.00) | 4761(1.00) | 0 | 1.000 |
|  |  | Po | 15(0.00) | 14(0.00) |  |  |
|  | Urine WBC | Ne | 3796(0.79) | 3749(0.79) | 1.335812 | 0.248 |
|  |  | Po | 979(0.21) | 1026(0.21) |  |  |
| gynecological examination | BV | Ne | 4696(0.98) | 4726(0.99) | 6.659572 | 0.010 |
|  |  | Po | 79(0.02) | 49(0.01) |  |  |
|  | CDV | 1 | 901(0.19) | 1023(0.21) | 61.69016 | <0.001 |
|  |  | 2 | 2962(0.62) | 3115(0.65) |  |  |
|  |  | 3 | 773(0.16) | 526(0.11) |  |  |
|  |  | 4 | 139(0.03) | 111(0.02) |  |  |
|  | VYI | Ne | 4531(0.95) | 4581(0.96) | 5.745234 | 0.017 |
|  |  | Po | 244(0.05) | 194(0.04) |  |  |

ALB: Serum albumin; ALT: Alanine transaminase; AST: Aspartate transaminase; BA: Basophil granulocytes; BG: Blood group; BIL: Urine bilirubin; Blood RH: Blood RH; BV: Bacterial vaginosis; Ca: Total calcium; CDV: Cleaning degree of vagina; CHOL: Total cholesterol; Cr: Creatinine; DB: Direct bilirubin; DBP: Diastolic blood pressure; EOS: Eosinophil granulocytes; FHR: Fetal heart rate; GLOB: Globulins; Glu: Plasma glucose (fasting); Hb: Hemoglobin; HCT: Hematocrit; IP: Serum inorganic phosphorus; KET: Urine ketone bodies; LY: Lymphocytes; MCH: Mean cell hemoglobin; MCHC: Mean corpuscular hemoglobin concentration; MCV: Mean cell volume; Mg: Magnesium; MID: Intermediate cell; MO: Monocytes; MPV: Mean platelet volume; NE: Neutrophil granulocytes; PCT: Plateletcrit; PDW: Platelet distribution width; P-LCR: Mean platelet volume; Plt: Platelet count; RBC: Red blood cells; RDW-CV: Red blood cell distribution width-CV; RDW-SD: Red blood cell distribution width-CV; SBP: Systolic blood pressure; TB: Total bilirubin; TBA: Total biliary acid; TG: Triglycerides; TP: Total protein; TSI: Total serum iron; UA: Uric acid; Urea: Urea; Urine WBC: Urine white blood cell; USG: Urine specific gravity; VYI: Vaginal yeast infection; WBC: White blood cell count; PTB: preterm birth

Table S4. Prenatal testing of pregnant women before 26 weeks of gestation between PTB group and control group

| Variables |  |  | Control (4775) | Case (4775) | t/chi | *P* |
| --- | --- | --- | --- | --- | --- | --- |
| physical examination | Waist size, cm | | 81.46±14.47 | 82.14±13.95 | -2.34503 | 0.019 |
|  | Fundal height, cm | | 19.77±3.55 | 20.11±3.73 | -4.54001 | <0.001 |
|  | SBP, mmHg | | 112.18±10.47 | 113.26±10.43 | -5.03821 | <0.001 |
|  | DBP, mmHg | | 69.52±7.79 | 70.67±16.28 | -4.43258 | <0.001 |
|  | FHR, times/min | | 145.78±3.09 | 146.68±19.22 | -3.21881 | 0.001 |
|  | Weight, Kg | | 62.68±9.19 | 62.56±9.28 | 0.650132 | 0.516 |
|  | Edema | Ne | 13(0.00) | 21(0.00) | 1.446326 | 0.229 |
|  |  | Yes | 4762(1.00) | 4754(1.00) |  |  |
| blood test | BG | A | 1238(0.26) | 1063(0.22) | 128.7004 | <0.001 |
|  |  | B | 1571(0.33) | 2107(0.44) |  |  |
|  |  | AB | 484(0.10) | 416(0.09) |  |  |
|  |  | O | 1482(0.31) | 1189(0.25) |  |  |
|  | Blood RH | Ne | 24(0.01) | 15(0.00) | 1.647755 | 0.199 |
|  |  | Po | 4751(0.99) | 4760(1.00) |  |  |
|  | ALB, g/L |  | 41.29±3.46 | 41.49±2.73 | -3.11588 | 0.002 |
|  | ALT, U/L |  | 20.75±14.26 | 21.08±13.78 | -1.15874 | 0.247 |
|  | AST, U/L |  | 20.95±7.87 | 22.17±7.73 | -7.62048 | <0.001 |
|  | Glu, mmol/L | | 4.61±0.55 | 4.50±0.45 | 10.33064 | <0.001 |
|  | Ca, mmol/L | | 2.30±0.13 | 2.31±0.11 | -1.65196 | 0.099 |
|  | Cr, umol/L |  | 50.86±7.62 | 51.18±8.25 | -1.93709 | 0.053 |
|  | DB, umol/L | | 1.76±0.95 | 1.72±0.72 | 2.214883 | 0.027 |
|  | TSI, umol/L | | 17.45±3.33 | 17.62±2.61 | -2.78362 | 0.005 |
|  | GLOB, g/L |  | 27.30±3.32 | 27.25±2.43 | 0.894102 | 0.371 |
|  | Mg, mmol/L | | 0.86±0.13 | 0.87±0.08 | -4.14576 | <0.001 |
|  | IP, mmol/L | | 1.25±0.15 | 1.25±0.12 | -1.70121 | 0.089 |
|  | TBA, umol/L | | 5.29±4.10 | 4.71±2.80 | 8.023786 | <0.001 |
|  | TB, umol/L | | 11.29±3.54 | 11.19±2.63 | 1.708969 | 0.087 |
|  | CHOL, mmol/L | | 4.74±0.71 | 4.76±0.36 | -1.30252 | 0.193 |
|  | TP, g/L |  | 68.83±4.84 | 68.84±3.52 | -0.06705 | 0.947 |
|  | TG, mmol/L | | 1.48±0.52 | 1.54±0.38 | -6.07971 | <0.001 |
|  | Urea, mmol/L | | 2.86±0.68 | 2.81±0.62 | 3.861467 | <0.001 |
|  | UA, umol/L | | 199.52±40.21 | 198.06±39.23 | 1.802698 | 0.071 |
|  | BA, 10e9L |  | 0.01±0.03 | 0.01±0.05 | -1.20236 | 0.229 |
|  | Plt, 10e9L |  | 220.91±48.42 | 224.66±48.28 | -3.79463 | <0.001 |
|  | EOS, 10e9L | | 0.09±0.09 | 0.09±0.07 | 0.03378 | 0.973 |
|  | Hb, g/L |  | 118.34±8.69 | 117.95±8.64 | 2.202925 | 0.028 |
|  | MID, 10e9L | | 0.54±0.10 | 0.55±0.11 | -4.19546 | <0.001 |
|  | LY, 10e9L |  | 1.72±0.40 | 1.75±0.41 | -2.91984 | 0.004 |
|  | MCH, pg |  | 31.46±1.91 | 31.31±1.84 | 3.943998 | <0.001 |
|  | MCHC, g/L | | 345.07±10.49 | 343.57±10.60 | 6.945442 | <0.001 |
|  | MCV, fL |  | 91.18±4.71 | 91.14±4.49 | 0.453407 | 0.650 |
|  | MO, 10e9L | | 0.53±0.14 | 0.54±0.14 | -4.12957 | <0.001 |
|  | MPV, fL |  | 8.58±1.11 | 8.60±1.09 | -0.9501 | 0.342 |
|  | NE, 10e9L |  | 7.22±1.69 | 7.34±1.72 | -3.40589 | <0.001 |
|  | P-LCR, % |  | 0.23±0.05 | 0.23±0.04 | 5.92923 | <0.001 |
|  | HCT, % |  | 0.34±0.02 | 0.35±0.25 | -1.10221 | 0.270 |
|  | PCT, % |  | 0.19±0.04 | 0.19±0.03 | 0.287164 | 0.774 |
|  | PDW, % |  | 15.13±2.28 | 14.81±2.51 | 6.579064 | <0.001 |
|  | RDW-CV, % | | 0.17±0.58 | 0.16±0.35 | 0.950871 | 0.342 |
|  | RDW-SD, fL | | 42.85±2.44 | 43.46±2.09 | -13.073 | <0.001 |
|  | RBC, 10e12L | | 3.77±0.32 | 3.78±0.32 | -0.83159 | 0.406 |
|  | WBC, 10e9L | | 9.57±1.93 | 9.72±1.96 | -3.74714 | <0.001 |
| urine test strip | Urine pH |  | 6.68±0.47 | 6.74±0.47 | -6.67754 | <0.001 |
|  | USG |  | 1.02±0.01 | 1.02±0.00 | 5.338421 | <0.001 |
|  | BIL | Ne | 4743(0.99) | 4753(1.00) | 1.50853 | 0.219 |
|  |  | Po | 32(0.01) | 22(0.00) |  |  |
|  | Glycosuria | Ne | 3906(0.82) | 3949(0.83) | 1.265278 | 0.261 |
|  |  | Po | 869(0.18) | 826(0.17) |  |  |
|  | KET | Ne | 4613(0.97) | 4607(0.96) | 0.078469 | 0.779 |
|  |  | Po | 162(0.03) | 168(0.04) |  |  |
|  | Nitrituria | Ne | 4729(0.99) | 4743(0.99) | 2.184509 | 0.139 |
|  |  | Po | 46(0.01) | 32(0.01) |  |  |
|  | Blood | Ne | 4338(0.91) | 4419(0.93) | 8.801462 | 0.003 |
|  |  | Po | 437(0.09) | 356(0.07) |  |  |
|  | Proteinuria | Ne | 4734(0.99) | 4718(0.99) | 2.319723 | 0.128 |
|  |  | Po | 41(0.01) | 57(0.01) |  |  |
|  | Bilirubinuria | Ne | 4759(1.00) | 4757(1.00) | 0.029517 | 0.864 |
|  |  | Po | 16(0.00) | 18(0.00) |  |  |
|  | Urine WBC | Ne | 3609(0.76) | 3558(0.75) | 1.397919 | 0.237 |
|  |  | Po | 1166(0.24) | 1217(0.25) |  |  |
| gynecological examination | BV | Ne | 4687(0.98) | 4721(0.99) | 7.784767 | 0.005 |
|  |  | Po | 88(0.02) | 54(0.01) |  |  |
|  | CDV | 1 | 884(0.19) | 1001(0.21) | 60.15999 | <0.001 |
|  |  | 2 | 2931(0.61) | 3092(0.65) |  |  |
|  |  | 3 | 804(0.17) | 555(0.12) |  |  |
|  |  | 4 | 156(0.03) | 127(0.03) |  |  |
|  | VYI | Ne | 4513(0.95) | 4561(0.96) | 4.884199 | 0.027 |
|  |  | Po | 262(0.05) | 214(0.04) |  |  |

ALB: Serum albumin; ALT: Alanine transaminase; AST: Aspartate transaminase; BA: Basophil granulocytes; BG: Blood group; BIL: Urine bilirubin; Blood RH: Blood RH; BV: Bacterial vaginosis; Ca: Total calcium; CDV: Cleaning degree of vagina; CHOL: Total cholesterol; Cr: Creatinine; DB: Direct bilirubin; DBP: Diastolic blood pressure; EOS: Eosinophil granulocytes; FHR: Fetal heart rate; GLOB: Globulins; Glu: Plasma glucose (fasting); Hb: Hemoglobin; HCT: Hematocrit; IP: Serum inorganic phosphorus; KET: Urine ketone bodies; LY: Lymphocytes; MCH: Mean cell hemoglobin; MCHC: Mean corpuscular hemoglobin concentration; MCV: Mean cell volume; Mg: Magnesium; MID: Intermediate cell; MO: Monocytes; MPV: Mean platelet volume; NE: Neutrophil granulocytes; PCT: Plateletcrit; PDW: Platelet distribution width; P-LCR: Mean platelet volume; Plt: Platelet count; RBC: Red blood cells; RDW-CV: Red blood cell distribution width-CV; RDW-SD: Red blood cell distribution width-CV; SBP: Systolic blood pressure; TB: Total bilirubin; TBA: Total biliary acid; TG: Triglycerides; TP: Total protein; TSI: Total serum iron; UA: Uric acid; Urea: Urea; Urine WBC: Urine white blood cell; USG: Urine specific gravity; VYI: Vaginal yeast infection; WBC: White blood cell count; PTB: preterm birth
